# A cluster-randomized controlled trial to evaluate the effects of a simplified cardiovascular management program in Tibet, China and Haryana, India: study design and rationale

**DOI:** 10.1186/1471-2458-14-924

**Published:** 2014-09-06

**Authors:** Vamadevan S Ajay, Maoyi Tian, Hao Chen, Yangfeng Wu, Xian Li, Danzeng Dunzhu, Mohammed K Ali, Nikhil Tandon, Anand Krishnan, Dorairaj Prabhakaran, Lijing L Yan

**Affiliations:** CoE-CARRS, Public Health Foundation of India, 4th Floor, Plot No – 44, Sector 44, Gurgaon, Haryana 122002 India; The George Institute for Global Health at Peking University Health Science Center, Suite 1801, Tower B, Horizon Tower, No. 6 Zhichun Road, Haidian District, Beijing, 100088 China; Department of Cardiology, Beijing Hospital, No. 1 Dahua Road, Dongcheng District, Beijing, 100730 China; Department of Epidemiology and Biostatistics, Peking University School of Public Health, No. 38 Xueyuan Road, Haidian District, Beijing, 100083 China; Tibet University, Jiangsu Road, Chengguan District, Lhasa, 850000 China; Rollins School of Public Health, Emory University, 1518 Clifton Road, Atlanta, GA 30322 USA; Department of Endocrinology and Metabolism, All India Institute of Medical Sciences, Ansari Nagar, New Delhi, 110029 India; Department of Community Medicine, All India Institute of Medical Sciences, Ansari Nagar, New Delhi, 110029 India; Centre for Chronic Disease Control, 4th Floor, Plot # 47, Sector-44, Gurgaon, New Delhi, 122002 India; Duke Global Health Institute, and Global Heath Research Center, Duke Kunshan University, China No.1699 Zuchongzhi Road, Kunshan, Jiangsu Province 215347 China

**Keywords:** Cardiovascular diseases prevention and control, Research design, Developing countries, Rural population, Community health workers, Risk reduction, Electronic decision support system

## Abstract

**Background:**

In resource-poor areas of China and India, the cardiovascular disease burden is high, but availability of and access to quality healthcare is limited. Establishing a management scheme that utilizes the local infrastructure and builds healthcare capacity is essential for cardiovascular disease prevention and management. The study aims to develop, implement, and evaluate the feasibility and effectiveness of a simplified, evidence-based cardiovascular management program delivered by community healthcare workers in resource-constrained areas in Tibet, China and Haryana, India.

**Methods/design:**

This yearlong cluster-randomized controlled trial will be conducted in 20 villages in Tibet and 20 villages in Haryana. Randomization of villages to usual care or intervention will be stratified by country. High cardiovascular disease risk individuals (aged 40 years or older, history of heart disease, stroke, diabetes, or measured systolic blood pressure of 160 mmHg or higher) will be screened at baseline. Community health workers in the intervention villages will be trained to manage and follow up high-risk patients on a monthly basis following a simplified ‘2 + 2’ intervention model involving two lifestyle recommendations and the appropriate prescription of two medications. A customized electronic decision support system based on the intervention strategy will be developed to assist the community health workers with patient management. Baseline and follow-up surveys will be conducted in a standardized fashion in all villages. The primary outcome will be the net difference between-group in the proportion of high-risk patients taking antihypertensive medication pre- and post-intervention. Secondary outcomes will include the proportion of patients taking aspirin and changes in blood pressure. Process and economic evaluations will also be conducted.

**Discussion:**

To our knowledge, this will be the first study to evaluate the effect of a simplified management program delivered by community health workers with the help of electronic decision support system on improving the health of high cardiovascular disease risk patients. If effective, this intervention strategy can serve as a model that can be implemented, where applicable, in rural China, India, and other resource-constrained areas.

**Trial registration:**

The trial was registered in the clinicaltrials.gov database on 30 December, 2011 and the registration number is NCT01503814.

## Background

An estimated 17 million people die of cardiovascular disease (CVD) each year with over 80% of these deaths occurring in low and middle-income countries [1]. Resource-poor areas in China and India, where CVD is already the leading cause of death, confront a rising disease burden with a strained healthcare system often inadequately equipped to deal with these problems [2–4]. Many of the risk factors strongly associated with CVD, including high blood pressure, tobacco use, and poor diet are among the top risk behaviors in these countries [5, 6]. For example, in studies assessing hypertension in rural areas of Tibet, China, up to 14.9% of the population aged 15 years or older was found to be hypertensive, with some townships having rates as high as 55.9% in adults 40 years old and above [7, 8]. The overall hypertension rate was found to be just as high in Haryana, India, with the urban slums of Faridabad District, Haryana similarly affected at 16.5% [9, 10]. Despite these high rates of hypertension, the number of patients receiving antihypertensive therapy in these areas remains low, and way below the averages seen in urban areas [7, 11].

There are many well-established interventions such as lifestyle modification of known risk factors and effective, low-cost medications that can help avert the CVD burden in these economic and healthcare resource limited settings [12–21]. One particularly cost-effective approach is to identify and manage individuals at high cardiovascular disease risk in order to prevent or delay events [22–28]. This method has been applied and implemented in the Rural Andhra Pradesh Cardiovascular Prevention Study in India and the China Rural Health Initiative in northern China [29–31].

Community Health Workers (CHWs) are the members who received basic professional training to provide health and medical care for the community. Involvement of CHWs in disadvantaged populations has resulted in a significant improvement in accessing healthcare facilities, screening disease, monitoring community members’ health status and adherence to treatment [32–35]. Task-shifting to CHWs has been proven as a cost-effective and sustainable approach in chronic disease prevention and control, particularly in those areas with limited economic and healthcare resources [36]. More recently, with the aid of mobile phones, studies have shown a significant reduction in CHW training time, disease screening time and an enhanced user satisfaction [37]. A recent systematic review also shows that mobile phone based technology in low and middle income countries positively impacts the chronic disease management and its associated clinical outcomes [38].

Simplifying and extending the high-risk intervention strategy to suit areas with high CVD burden, but more limited economic and healthcare resources will be essential to addressing this problem in an effective and affordable way in these areas. China and India are two largest developing countries in the world that face similar challenges in terms of the burden of CVD and resource constraints in tackling these challenges. Yet, there are also many differences in the healthcare systems, socio-economical environment and ethno-cultural customs that will make conducting a joint study illuminating and fruitful.

Therefore, we have designed a study to develop and evaluate the effects of implementing, in resource-scarce settings, a highly **sim**plified but guideline-based program for **card**iovascular management by CHWs with the aid of electronic decision support system in rural China and India (the SimCard study). This paper describes the research design and the rationale of the study.

## Methods/design

The SimCard study is a cluster-randomized controlled interventional trial to be conducted in rural areas of Tibet, China and Haryana, India. The design, implementation and reporting of the study follow recommendations from CONSORT statement on cluster-randomized trials [39]. The trial was registered in the clinicaltrials.gov database on 30 December, 2011 and the registration number is NCT01503814. The study protocol received ethics approval from the institutional review boards at Peking University Health Sciences Center, China, the Public Health Foundation of India, India, and Duke University, USA. Informed consent will be obtained from each study participant.

### Study sites

Two counties in the Tibet autonomous region located in Southwest China – Gongbujiangda and Linzhou - and one tehsil - Ballabgarh in Faridabad District from Haryana State in northwestern India will participate in the study. Villages from these locations will be selected to take part in this study based on the site selection criteria of (1) high CVD burden, (2) limited healthcare resources, (3) having available existing CHWs or qualified candidates who can be trained to fulfill that role, and (4) local government support. A total of 40 villages will be recruited across the two countries, 20 in each country.

### Randomization

In each country, villages will be randomized to receive the intervention or usual care. Randomization will be stratified by country. In China, all selected villages will be randomized by a 1:1 ratio to the intervention and control group with stratification by county and township (the administrative unit managing the villages). In India, randomization will not be stratified by any geographical locations. Randomization in both countries will be conducted by a study staff not involved in the intervention through a central computerized process. Group allocation will be concealed until after the baseline data collection is completed.

### Study population

Participants at CVD high risk will be eligible for study enrollment. In this study, ‘high-risk’ is defined as older age (40 years old and above) with a history of any of the following conditions: coronary heart disease, stroke (ischemic, hemorrhagic, or unknown/unspecified type), diabetes mellitus, and/or having a systolic blood pressure ≥160 mmHg at two different time points measured in the same day during the baseline survey.

Exclusion criteria include having CVD-related complications that cannot be managed in a primary care setting, having a malignancy or life-threatening disease; individuals who are bed-ridden, currently participating in any other clinical trials, unable to stay in the village longer than 8 months in a year, and planning to move in the next year.

### Screening

Before the start of the baseline data collection, a survey will be conducted in all participating villages to screen for high-risk individuals. All village residents 40 years and above will be invited to participate in the survey, which will include a brief questionnaire on disease history and measuring blood pressure twice. All high-risk individuals screened out through this process according to the definition described above, a target minimum of 50 in each village, will be invited to participate in the study.

### Intervention and control

#### Intervention

The intervention scheme adapts and simplifies international and applicable national clinical guidelines for hypertension and cardiovascular disease management for use by local CHWs to manage cardiovascular high-risk patients in an easily implementable, low cost, and medically effective way [23, 40]. Local CHWs will be trained to provide basic monthly follow-up care to identified high-risk patients, and refer them to higher level healthcare facilities when necessary. The intervention strategy consists of the following:

#### ‘2 + 2’ intervention model

Taking into consideration the local health behaviors and resources, a simplified management model is devised that focuses on two therapeutic lifestyle recommendations (smoking cessation and salt reduction) and the appropriate prescription of two types of accessible, highly effective, generally safe, and low-cost drugs (blood pressure lowering drugs and aspirin) [41–46]. In India, as stipulated by the ethic committee, the drugs used in the intervention arm will be also made available to the primary care facilities serving the control villages. Coined the "2 + 2" intervention model, the scheme targets two prevalent risk behaviors with established relationships to CVD while providing instruction on the prescription of two types of basic drugs that have well-documented effects on CVD risk reduction. All individuals in the intervention group will receive therapeutic lifestyle recommendations when applicable, but depending on each individual’s medical situation may be prescribed both, one, or none of the two types of drugs. These interventions will be delivered by CHWs and they will provide monthly follow-up to all high-risk patients under their care.

#### Systematic training of local CHWs

Enhancing the capacity of local CHWs through systematic training is a key feature of the intervention model. Training of local CHWs in the intervention group will consist of an initial training on the intervention protocol, including education on the targeted CVD lifestyle risk factors and the medications being utilized, followed by refresher training about 1–3 months after the intervention begins. Only CHWs who pass the initial evaluation examination can take part in implementing the intervention. After the training, CHWs will be provided with a list of high-risk individuals screened out at baseline for them to contact and manage according to the protocol.

In Tibet, China, "village doctors" who are not physicians but have basic professional training and can prescribe medications will be chosen to be the CHWs to implement this management plan. In rural India, local women will be recruited and trained to be the study’s CHWs. Because the Indian CHWs have no prior medical experience, they will be partnered with licensed physicians from nearby primary health care centers who are responsible for the prescription duties based on the CHWs’ regular assessments of their assigned high-risk patients.

#### Electronic decision support

An electronic decision support (EDS) component will be incorporated in this study to assist the CHWs on the follow up and management of their high-risk patients during the one-year intervention period. The EDS system is a smartphone or tablet-based android application that will be developed and pilot tested based on the ‘2 + 2’ intervention model and consists of prompts regarding the patient’s medical history, new conditions, medication usage, current lifestyle habits, blood pressure, and the appropriateness for prescribing any of the target medications. The follow-up records entered into the device can be easily uploaded by CHWs to a central server to generate performance indicators and provide feedback to help improve the quality of care. In India, the EDS system has a desktop component for the use of physicians to prescribe drugs in the intervention arm.

#### Performance feedback

Tiered monetary payments based on key performance indicators will be given to CHWs on a recurring basis. Examples of key performance indicators include the number and percentage of high-risk individuals receiving regular follow-up, lifestyle advices, and medications.

#### Control

Villages in the control group will continue their usual practices without any of the above mentioned interventions. Usual practices will include any other government or project-initiated lifestyle, hypertension or cardiovascular management programs.

### Outcome evaluation

#### Baseline and post-intervention survey

The outcome evaluation for this study will be derived from data collected from the baseline survey and post-intervention follow-up survey of all eligible and consenting high-risk patients in the intervention and control villages. Both survey questionnaires will be identical, and include information on patient’s demographics, disease history, lifestyle behaviors, medication use, primary healthcare services utilization, hospitalizations, and medical care related expenses in the past year. Height, weight, waist circumference, and blood pressure will be measured at that time of the survey. Surveys in both countries will be conducted by trained personnel according to the same standardized operating procedures. The questionnaires used in China and India will be kept nearly identical except for a few variations to fit the cultural context and target the specific lifestyles of the two peoples in these countries such as different types of tobacco use (See Table 1 for survey indicators).

**Table 1:** **Indicators followed in baseline and follow-up survey**

Indicators	Measures	Methods
Behavioral	Tobacco	Questionnaire
	Alcohol*	
	Physical activity	
	Salt consumption	
Anthropometric	Height	Stadiometer
	Weight	Weighing machine
	Waist circumference	Measuring tape
	Blood pressure	Electronic BP monitor
Treatment history, Health services, Health care costs	Compliance	Questionnaire
	Awareness, Risk factor control	
	Service utilization	
	Treatment cost	

*: Not covered in Tibet, China site.

The primary outcome will be the net difference between the intervention and control groups in the pre- and post-intervention change in the proportion of high-risk individuals treated with anti-hypertensive medication, obtained through the two surveys described above. This process indicator is chosen for its close association with the intervention scheme, effect on lowering high blood pressure, and its excellent power.

A number of secondary outcomes will also be evaluated, including the net difference between the intervention and control groups in the pre- and post-intervention change in the proportion of high-risk individuals treated with aspirin, the net difference in mean blood pressure from baseline to follow-up in high-risk individuals between the intervention and control villages, as well as hypertension awareness, treatment, and control rates. Awareness of modifiable lifestyle risk behaviors will also be assessed, specifically on the proportion of high-risk individuals aware of the harms of smoking and consumption of a high salt diet. Finally, the proportion of high-risk individuals receiving five or more follow-up visits from CHWs will also be calculated.

### Sample size

Key underlying assumptions for this study are that there will be 20 intervention villages and 20 control villages with 50 consenting high-risk patients in each village and an estimated total of 2,000 high-risk individuals, an intra-cluster correlation coefficient (ICC) of 0.01 or 0.02 [36, 47], and two-sided alpha of 0.05. For the primary outcome, assuming the proportion of anti-hypertensive medication prescriptions in the control villages is 20% (conservative as preliminary data shows it to be <10% in the target study sites), the power to detect a 10% difference in the primary outcome of anti-hypertensive medication use is excellent (>90%) with an ICC of 0.01, and similarly high with an ICC of 0.02. Assuming a standard deviation of change in systolic blood pressure of 15 mmHg among the high-risk individuals, the power to detect a 3 mmHg net difference in this secondary outcome between the intervention and control group pre-post differences will be >90% with an ICC of 0.01 (>90% as well if ICC = 0.02).

### Statistical analysis

All analyses will be conducted at individual level according to intention-to-treat principles, and take into account the stratification design and cluster effects. For the primary outcome, anti-hypertensive medication use, log-binomial models with random effects for villages to account for clustering and random effects for intercept to account for the pre-post correlations within individuals and a fixed effect for country to account for the stratified study design will be used. Other than the main effect from intervention and time, an interaction term between intervention and time will be added in the model to test if the slope of the pre-post changes differs by intervention or control group. To account for the heterogeneity between two countries, the two-way interaction of the country variable with intervention and time will be included into the above models. In the case of significant interaction existing, the separate analysis by country will be conducted afterward. The net effect from intervention will be reported as difference-in-difference of proportions together with model-based 95% confidence intervals obtained by transforming back from models. For continuous outcomes, such as systolic blood pressure, similar strategies will be adopted but linear models instead of log-binomial models will be used. Some adjusted analysis will also be performed to account for potential unbalanced baseline information. Other pre-specified subgroup will include analyses by sex and age (40–59 vs. 60 years or older) using similar strategy for the country variable.

### Process and economic evaluation

Process evaluation will be conducted at the end of the intervention program to investigate the extent to which the intervention has been implemented as designed, and to identify the facilitators and barriers to the implementation of the intervention program at each level of the study. We will conduct face-to-face, semi-structured interviews by independent interviewers. Interviewees include the CHWs, high-risk individuals in the selected villages, local project administrators, and government officials. A standardized interview guideline will be developed that contains a mixture of close- and open-ended questions. All interviews will be audio-recorded, transcribed and analyzed using a qualitative descriptive interpretive approach combing thematic content analysis and constant comparison methods facilitated by QSR NVIVO 10.0 data management software. The economic evaluation will help determine the economic feasibility of expanding such an intervention using data collected on healthcare services usage and medication use as obtained from the follow-up surveys.

## Discussion

CVD is a global problem that affects developed and developing countries alike. Areas with constrained economic or healthcare resources face an even tougher challenge as the awareness of CVD is lacking and trained healthcare professionals are limited. The SimCard study in China and India aims to evaluate a simplified, multifaceted, and innovative intervention scheme for CVD prevention and control in resource-scarce settings. To the best of our knowledge, SimCard is the first study to incorporate an EDS system delivered by the CHWs for CVD management in these two countries. The findings of the study will provide evidence on the feasibility, acceptability and effectiveness of a simplified yet guideline-based intervention program delivered by the CHWs in resource constrained areas.

The study design aims to maximize the feasibility of the intervention through a simplified culturally-tailored CVD prevention and management plan based on the guidelines. Despite there being well-established national guidelines and policies on effective strategies to prevent and control CVD in China and India, its uptake into routine medical care remains very limited in resource constrained areas. This is primarily due to the complexity of the guideline, the lack of awareness of the guidelines by the CHWs and CHW’s limited capacity to implement the guidelines. CHWs who will be utilizing the simplified management scheme descried in this study will be thoroughly trained and tested for competency on the indications, contraindications and side effects of the medications. CHWs will also be trained to provide therapeutic lifestyle recommendations as applicable. CHWs will be provided incentives based on the key performance indicators such as the regular follow-up rate, the percentage of high-risk individuals receiving lifestyle advices and other measurements.

The intervention is also designed to be innovative. By 2013, there were over 2 billion mobile phone subscribers in China and India [48]. This accounts for a staggering one third of the world’s mobile users [48]. Smartphones and tablets with their advanced multimedia capability and connectivity to the internet are rapidly expanding in the developing countries’ market. These mobile devices are rapidly becoming the dominant mode of accessing the internet and for example, China represents 22% of the global smartphone subscribers [49]. China has surpassed the U.S.A. to become the world’s top country for active Android and iOS users. Given the near ubiquity of mobile phones and the exponential growth of smart phones and tablets in Chinese and Indian markets, these devices represent one of the few hardware products available with the potential to transform the delivery of health care. There is growing evidence showing smartphone or tablet based EDS system can improve patient care in both developed and developing countries [38, 50]. Therefore, we incorporate the smartphone or tablet based EDS system into the intervention aiming to help the CHWs better follow-up and manage their high CVD risk patients.

Such a study is needed in both rural China and India where the prevalence of hypertension and CVD has been increasing rapidly and has reached epidemic proportions. The healthcare systems in both countries by and large are still acute care-oriented and ill-equipped to tackle these challenges. Shifting to and sharing of certain preventive tasks with CHWs is one important strategy that both countries need to evaluate for effectiveness and future scale-up if possible. In addition, implementation of the intervention needs to adapt to local contexts in order to be successful. China and India (in our study, Tibet and Haryana, respectively) possess distinctive features in primary healthcare delivery models, local cultural values and practices, and even regulations and governance of research studies. We anticipate this joint study in two countries will shed light on how this adaptation process can take place and how these differences may influence the results of the study. For example, in Tibet, Tibetan medicine is a widely believed system that can sometimes be at odds with Western medicine. In north India, women do not generally interact with non-family men. This gender relations issue will potentially pose some constraints to the Indian CHWs, who will all be female, in carrying out the management plan among male high-risk patients, which can be addressed with involvement of women from the patient’s household. Many strategies and standardized operation procedures will be in place to ensure the quality of the implementation of the study protocol while taking into consideration differences between the two countries.

The burden of non-communicable chronic diseases is rapidly rising, but many areas lack the economic and healthcare resources to effectively deal with these widespread health problems. While evidence-based national and international guidelines on managing chronic conditions such as hypertension, coronary heart disease, and stroke are well-established, cost-effective approaches suitable for adoption in resource-constrained settings have not been adequately investigated. The SimCard study aims to address the highly prevalent problem of CVD in these remote and resource- limited areas that have not received much attention so far. The results of the study are expected both to advance scientific knowledge and to provide translational evidence necessary for sound policy making to address the CVD problem in these settings, in China, India, and where applicable, to other countries.

## Abbreviations

CVD: Cardiovascular disease
CHW: Community health worker
EDS: Electronic decision support.
